# Mikronährstoffbedarfe im Sport

**DOI:** 10.1007/s00103-025-04132-3

**Published:** 2025-10-07

**Authors:** Anja Carlsohn

**Affiliations:** Fakultät Gesundheit, Hochschule für Angewandte Wissenschaften Hamburg, Ulmenliet 20, 21033 Hamburg, Deutschland

**Keywords:** Mikronährstoffe, Athleten, Gesundheit, Leistungsfähigkeit, Sportassoziierte Ernährungsrisiken, Micronutrients, Athletes, Health, Performance, Sports-associated nutritional risks

## Abstract

Das narrative Review gibt einen literaturbasierten Überblick über Mikronährstoffbedarfe im Sport. Dabei werden potenziell erhöhte Bedarfe, erhöhte Verluste sowie Effekte von Mikronährstoffdefiziten auf Gesundheit und Leistungsfähigkeit berücksichtigt.

Sporttreibende mit einer energiebedarfsdeckenden Ernährung sind in der Regel ausreichend mit Mikronährstoffen versorgt, jedoch können ungünstige Ernährungsweisen in Kombination mit erhöhten Verlusten z. B. über den Schweiß zu Mikronährstoffdefiziten führen. Risiken für Defizite bestehen v. a. bei restriktiver Energiezufuhr, einseitiger Ernährung und hoher Trainingsbelastung. Zu den kritischen Nährstoffen bei Sporttreibenden können je nach individueller Ernährungs- und Trainingsbelastung die Mineralstoffe Eisen, Natrium, Zink und Calcium sowie Vitamin D gehören.

Die Diagnostik eines Mikronährstoffdefizits sollte laborgestützt erfolgen. Eine ausgewogene, energiebedarfsdeckende Ernährung mit nährstoffschonenden Lager- und Zubereitungsverfahren nach dem Food-First-Prinzip (d. h. bedarfsdeckende Lebensmittelzufuhr ist gegenüber Nahrungsergänzungen zu priorisieren) ist die wichtigste Maßnahme, um eine bedarfsgerechte Mikronährstoffversorgung bei Sporttreibenden zu sichern.

## Abstract (en)

This narrative review provides a literature-based overview of micronutrient requirements in sports and exercise. It takes into account potentially increased requirements, increased losses and the effects of micronutrient deficiencies on health and performance.

Athletes with a diet that meets their energy requirements are generally adequately supplied with micronutrients, but unfavourable diets combined with increased losses, for example through sweat, can lead to micronutrient deficiencies. Risks of deficiencies exist primarily in cases of restrictive energy intake, an unbalanced diet and high training loads. Depending on individual diet and training load, critical nutrients for athletes may include the minerals iron, sodium, zinc and calcium as well as vitamin D.

The diagnosis of a micronutrient deficiency should be laboratory-based. A balanced diet that meets energy requirements with nutrient-preserving storage and preparation methods based on the food-first principle (i.e. food intake that meets requirements should be prioritised over dietary supplements) is the most important measure for ensuring that athletes receive the micronutrients they need.

## Einleitung

Mikronährstoffe, d. h. Vitamine und Mineralstoffe, sind für die Gesundheit und Leistungsfähigkeit Sporttreibender genauso essenziell wie für die moderat körperlich aktive Allgemeinbevölkerung. Bei einer energiebedarfsdeckenden Ernährungsweise – die bei Sporttreibenden hyperkalorisch verglichen mit moderat körperlich aktiven Personen ist – nimmt die Mikronährstoffzufuhr über die habituelle Ernährung aufgrund des gesteigerten Verzehrvolumens zu und führt in der Regel zu einem Erreichen bzw. Überschreiten der nationalen Referenzwerte für die Nährstoffzufuhr [1]. Allerdings kann es sportassoziiert zu erhöhten Bedarfen kommen, wobei zu differenzieren ist zwischen a) sportassoziierten erhöhten Verlusten (beispielsweise über den Schweiß), b) sportartspezifisch gehäuften Ernährungsrisiken (z. B. restriktive Ernährung in gewichtsensitiven Sportarten) und c) metabolisch bedingten Mehrbedarfen (z. B. am Energie- und Proteinstoffwechsel beteiligte Vitamine).

Diese Arbeit gibt eine narrative Übersicht über potenzielle sportassoziierte Mehrbedarfe, Risikokonstellationen für Mikronährstoffdefizite sowie aktuell gesicherte Effekte von sowohl Vitamin- oder Mineralstoffdefiziten als auch Mikronährstoffsupplementierungen auf Parameter von Gesundheit und sportlicher Leistungsfähigkeit.

## Sportassoziiert erhöhte Mikronährstoffverluste

Mit zunehmender sportlicher Betätigung steigt der Schweißverlust, wobei Sportler*innen mit dem Schweiß auch Mineralstoffe verlieren. Wie relevant Schwitzen den Bedarf bzw. den Versorgungszustand an bestimmten Mineralstoffen von Sporttreibenden beeinflusst, hängt u. a. wesentlich von der individuellen Schweißrate und dem Trainingsumfang sowie dem Mineralstoffgehalt des Schweißes ab, was interindividuell erheblich variieren kann [2]. Dies macht eine hinreichend valide Vorhersage, wie viel eines Mikronährstoffs beispielsweise nach 3 h Radfahren über den Schweiß verloren geht, unmöglich.

Bei Ausdauersportler*innen zeigt die Literatur Schweißraten zwischen 0,4 und 1,8 Litern pro Stunde, wobei der Mineralstoffgehalt im Schweiß individuell stark variiert. Insbesondere der Natriumanteil kann bis zu Faktor 10 schwanken [3]. Eine längere Belastungsdauer sowie eine fortschreitende Hitzeakklimatisierung reduzieren die im Schweiß enthaltenen Mineralstoffe.

Bei einer durchschnittlichen Schweißrate und sportartspezifisch moderatem bis hohem Belastungsumfang können über den Schweiß somit relevante Mineralstoffverluste von Natrium, Kupfer und Zink auftreten (Tab. 1). Auch der an sich marginale schweißbedingte Eisenverlust kann – in Kombination mit anderen sportinduzierten Verlusten bzw. entsprechenden Mehrbedarfen – durchaus zur Entwicklung eines Eisenmangels beitragen. Die Verluste weiterer Mineralstoffe über den Schweiß sind in der Regel so gering, dass sie als vernachlässigbar gelten [4].

**Tab. 1:** Geschätzte Mineralstoffverluste über den Schweiß [4] bei einer 70 kg schweren Person mit einer Belastungsintensität von 10 km/h (Laufen, 15 °C Außentemperatur). (Modifiziert nach [3] (für Calcium, Natrium, Kalium, Kupfer, Magnesium und Zink) bzw. nach [39] (für Eisen) und [40] (für die Schweißraten))

Mineralstoff	Mineralstoffkonzentration im Schweiß bei ca. 60-minütiger Belastung [mg/l] (interindividuelle Variation als Range in mg/l)	Geschätzter Verlust während 45 min Trainings bei einer Schweißrate von 0,8 l/Stunde [mg/Stunde]
Calcium	18 (11–36)	11
Eisen	0,56 (0–1,12)	0,34
Natrium	874 (175–1512)	524
Kalium	196 (167–236)	117
Kupfer	0,11 (0,04–0,22)	0,07
Magnesium	1,43 (0,84–2,36)	0,86
Zink	0,65 (0,29–1,23)	0,39

Ob und in welchem Ausmaß körperliche Aktivität mit Faktoren wie Trainingsintensität und -umfang die Mineralstoffausscheidung über Urin und/oder Fäzes beeinflusst, ist bislang nicht eindeutig geklärt. So zeigen ältere Studien zwar eine relevant erhöhte Ausscheidung von Magnesium oder Zink [5, 6], aktuell fehlt dafür aber die Evidenz.

Vitaminverluste über den Schweiß dagegen sind marginal. Die Vitamine C und B1 konnten zwar in kleinsten Mengen im Schweiß von Sporttreibenden nachgewiesen werden [7], dies spielt aber selbst bei Athlet*innen mit sehr hohen Schweißverlusten praktisch keine Rolle für den Versorgungszustand. Ein sportbedingter Mehrbedarf an Vitaminen infolge erhöhter Verluste über den Schweiß ist derzeit nicht anzunehmen.

## Sportartspezifische Ernährungsrisiken mit potenziell unzureichender Zufuhr an Mikronährstoffen

Insbesondere bei einer restriktiven, hypokalorischen Ernährung ist es unabhängig von der sportlichen Belastung schwierig, alle Mikronährstoffempfehlungen zu erreichen, selbst wenn gezielt Lebensmittel mit niedriger Energie- und hoher Mikronährstoffdichte ausgewählt werden.

Tatsächlich gelingt es Sporttreibenden nicht immer, sich isokalorisch und nährstoffbedarfsgerecht zu ernähren, da sportartspezifisch teilweise periodisch variierende Ernährungsziele verfolgt werden (z. B. akute Gewichtsreduktion, Carboloading, Train-Low-Techniken usw.). Restriktive Ernährungsweisen mit einem erhöhten Risiko für eine mangelhafte Versorgung mit Eisen und Calcium sind besonders in Sportarten verbreitet, in denen das Körpergewicht leistungsbestimmend ist (z. B. Ausdauer-, ästhetische oder technische Sportarten; [8]).

Die Lebensmittelauswahl von Athlet*innen kann sowohl dauerhaft aufgrund von Unverträglichkeiten oder aus ethischen, religiösen oder anderen persönlichen Gründen als auch zeitweise während Trainings- oder Wettkampfreisen, bei Aufenthalten in Höhencamps oder in gewichtsreduzierenden Phasen eingeschränkt sein [4]. Eine professionelle Ernährungsbetreuung von Sporttreibenden durch sportspezifisch qualifizierte, zertifizierte Ernährungsberater*innen ist empfehlenswert, um individuelle Lösungsansätze zu entwickeln.

Aufgrund der geringen Prävalenz veganer Kaderathlet*innen in Deutschland (ca. 0,3 %) und der folglich unzureichenden Studienlage lassen sich keine konkreten Aussagen treffen, ob eine vegane Ernährung bei Sportler*innen das Risiko für Mikronährstoffmängel erhöht oder mit gesundheits- und/oder leistungsbezogenen Vor- und Nachteilen zu rechnen ist [9]. Grundsätzlich ist bei veganen Sportler*innen von ähnlichem gesundheitlichen Nutzen des Sporttreibens, ähnlichen kritischen Nährstoffen hinsichtlich der Versorgung sowie einer analogen Supplementierungsempfehlung mit Vitamin B12 wie in der veganen Allgemeinbevölkerung auszugehen. Eine vegetarische Kostform scheint für Sportler*innen unproblematisch zu sein, sofern die Lebensmittelauswahl ausgewogen ist und eine regelmäßige Überprüfung kritischer Nährstoffe (z. B. der Eisenversorgung) durchgeführt wird [10].

Aufgrund des „Mengen-Zeit-Problems“ (d. h. hoher Energiebedarf bei geringen zeitlichen Ressourcen für Nahrungszubereitung, Nahrungszufuhr und Verdauung vor dem Training) erreichen Sportler*innen nicht immer die nationalen Empfehlungen für Gemüse und Obst [11]. Dadurch kann es beispielsweise zu einer kritischen Versorgung von Folat sowie einer geringeren Aufnahme sekundärer Pflanzeninhaltsstoffe kommen. Eine personalisierte Ernährungsberatung kann entsprechende Alternativen aufzeigen (z. B. den Verzehr gegarter, gut verträglicher Gemüsesorten, eine Anreicherung der Mahlzeiten mit Nüssen oder Saaten, die Integration von Gemüse- und Fruchtsäften in die Mahlzeiten, Verzehr von Gemüse- oder Obstpürees).

## Sportassoziierter metabolischer Mehrbedarf an Mikronährstoffen

Für das Verständnis, dass sportliche Aktivität nicht *per se* mit einem Mehrbedarf an Mikronährstoffen assoziiert ist, ist es hilfreich, sich zu vergegenwärtigen, dass Mikronährstoffe häufig als Co-Faktoren, Coenzyme oder Second Messenger in Signalkaskaden fungieren und nach „Gebrauch“ teilweise „recycelt“ oder über den enterohepatischen Kreislauf reabsorbiert werden können.

Calcium beispielsweise ist für die Muskelkontraktion unerlässlich, da es durch Bindung an Troponin und Freilegung der Myosin-bindenden Stellen auf dem Aktinfilament den myofibrillären Ruderschlag ermöglicht. Das Calcium ist jedoch nicht nach jeder Muskelkontraktion endgültig verbraucht, sondern gelangt wieder zurück in das sarkoplasmatische Retikulum, wo es bei Bedarf erneut freigesetzt werden kann.

Insbesondere bei Vitaminen, die am Energie- oder Proteinstoffwechsel beteiligt sind, ist jedoch ein Energie- bzw. Proteinumsatz-assoziierter Mehrbedarf gut belegt [4]. Zu den Vitaminen, die von einem erhöhten Bedarf betroffen sind, zählen Thiamin (Vitamin B1), Riboflavin (Vitamin B2) und Niacin. Bei einer energiebedarfsgerechten Ernährung stellt sich die Bedarfsdeckung jedoch als unproblematisch dar. Die Referenzwerte der Deutschen Gesellschaft für Ernährung (DGE) für diese Vitamine lassen sich auch bei einem gesteigerten Energieverbrauch aufgrund sportlicher Aktivität anwenden, da die Referenzwerte die Energiezufuhr berücksichtigen (Tab. 2). Bei Athleten*innen sollten demnach nicht die alters- und geschlechterspezifischen Referenzwerte herangezogen, sondern die energieumsatzabhängigen Werte berücksichtigt werden.

**Tab. 2:** Empfehlungen der Deutschen Gesellschaft für Ernährung für die Vitamine B1, B2 und Niacin für Sportler*innen in Abhängigkeit der Energiezufuhr [4]; d = Tag

Vitamin	Referenzwerte Männer/Frauen (25 bis < 51 Jahre)	Empfehlung bei Energiezufuhr von 2000 kcal/d	Empfehlung bei Energiezufuhr von 2500 kcal/d	Empfehlung bei Energiezufuhr von 3500 kcal/d
Vitamin B1 [mg/d]	1,2/1,0	1,1	1,4	1,9
Vitamin B2 [mg/d]	1,4/1,1	1,2	1,5	2,1
Niacin [mg/d]	15/12	13,2	16,5	23,1

Zusammenfassend haben sportassoziiert ungünstige Ernährungsverhaltensweisen einen erheblich größeren Einfluss auf den Versorgungszustand von Athlet*innen mit Mikronährstoffen als die häufig überschätzten „sportbedingten Mehrbedarfe“ durch Schweißverluste an Vitaminen und Mineralstoffen [12]. Eine bedarfsgerechte Lebensmittelauswahl sowie die ernährungskompetente Zubereitung von Lebensmitteln beeinflussen den Versorgungszustand von Athlet*innen mit Mikronährstoffen erheblich. Beispielsweise kann durch kompetente Wahl des Erhitzungsverfahrens der Mineralstoffgehalt in der Speise um 15–20 % und der Vitamingehalt um bis 50 % höher sein (z. B. Dampfgaren statt Kochen; [13]).

Sportler*innen, die infolge diverser Ursachen einen über dem Durchschnitt liegenden Bedarf an einzelnen oder mehreren Mikronährstoffen oder ein erhöhtes Risiko für eine unzureichende Versorgung aufweisen, sind eine isokalorische und ausgewogene Lebensmittelzusammenstellung sowie die nährstoffschonende Lagerung und Zubereitung anzuraten. Dieser Ansatz entspricht der Food-First-Strategie (d. h. Abdeckung der individuellen Bedarfe mit natürlichen Lebensmitteln priorisieren und Nahrungsergänzungsmittel meiden).

## Versorgungslage mit Mikronährstoffen bei Freizeit- und Spitzensportler*innen

Die Weltgesundheitsorganisation (WHO) empfiehlt zur Gesunderhaltung insgesamt 2,5 h pro Woche moderate bis intensive sportliche Aktivität im Ausdauerbereich sowie an mindestens 2 Tagen pro Woche Kräftigung der großen Muskelgruppen [14]. Die deutsche Bevölkerung, einschließlich Sporttreibender, die diese Empfehlungen erreicht, ist im Mittel gut mit Mikronährstoffen versorgt [15]. Sporttreibende zählen aufgrund ihres in der Regel höheren Energiebedarfs und höherer Verzehrmengen nicht zu den Risikogruppen für eine unzureichende Zufuhr von Vitaminen und Mineralstoffen.

Entgegen der verbreiteten Annahme, aufgrund der sportlichen Belastungen unzureichend mit Mikronährstoffen versorgt zu sein, zeigt die internationale Literatur im Mittel eine deutlich über die Empfehlungen hinausgehende Zufuhr von Vitaminen und Mineralstoffen bei Athlet*innen.

Bei 324 kanadischen Spitzensportler*innen mit geringfügig höherer Energiezufuhr als in der moderat aktiven Allgemeinbevölkerung (2920 ± 930 kcal/Tag (Männer) bzw. 2300 ± 700 kcal/Tag (Frauen)) wurde anhand von Verzehrprotokollen bei nahezu allen Mikronährstoffen eine deutlich die nationalen Empfehlungen überschreitende Versorgung allein aus Speisen und Getränken (d. h. ohne Nahrungsergänzungsmittel) dokumentiert [1]. Die mittlere Zufuhr variierte hierbei zwischen 120 % (für Calcium) und 365 % (für Vitamin C) der nationalen Zufuhrempfehlung für die gesunde Allgemeinbevölkerung. Nutzten diese Athlet*innen zusätzlich Nahrungsergänzungsmittel mit Mikronährstoffen, wurden die Zufuhrempfehlungen teilweise um das 8‑ bis 9‑Fache (z. B. Vitamin B6 und B12 bei männlichen Athleten) überschritten.

Die Sinnhaftigkeit einer zusätzlichen Supplementation bei mehr als empfehlungsgerechter Mikronährstoffzufuhr über Lebensmittel ist zu hinterfragen bzw. mit verschiedenen Risiken assoziiert (z. B. dopingrelevante Verunreinigungen, Wechselwirkungen mit Arzneimitteln, Überdosierungen, Verunreinigung mit toxischen oder nicht zugelassenen Substanzen; [16]).

Auch Wardenaar et al. zeigten in einer Studie mit 553 niederländischen Leistungssportler*innen, dass die Athlet*innen mit ihrer Basisernährung (d. h. ohne Nahrungsergänzungsmittel oder angereicherte Sportprodukte) ausreichend mit Calcium, Magnesium, Kupfer, Zink sowie den Vitamin E, B12 und Niacin versorgt waren [17]. Allerdings erreichte ein nennenswerter Anteil der untersuchten Athlet*innen die nationalen Zufuhrempfehlungen bei Vitamin A (20 % der Männer vs. 48 % der Frauen), Vitamin B1 (18 % vs. 40 %), B2 (12 % vs. 17 %) und Folsäureäquivalenten (15 % vs. 25 %) nicht.

Jedoch sollte kein Rückschluss von Personengruppen auf den Versorgungsstatus einzelner Athlet*innen gemacht werden, da unter bestimmten Risikokonstellationen (z. B. hypokalorische Kost, eingeschränkte Lebensmittelauswahl, Reisetätigkeit) durchaus eine individuell unzureichende Versorgung vorliegen kann.

Beispielsweise zeigt eine Untersuchung an deutschen Nachwuchsfußballerinnen, dass ein großer Anteil der Fußballerinnen die Referenzwerte für Calcium (59 %) und Eisen (69 %) nicht erreicht [18]. Eine unzureichende Mikronährstoffversorgung ist vor allem bei Nachwuchsathlet*innen verbreitet.

Betont werden muss allerdings auch, dass ein Nichterreichen der Referenzwerte für die Nährstoffzufuhr nicht mit einer defizitären Versorgung gleichzusetzen ist, da die Zufuhrempfehlung deutlich höher liegt als der durchschnittliche Bedarf. Damit werden Personen mit einem „überdurchschnittlich“ hohen Nährstoffbedarf – zum Beispiel Sporttreibende – bereits in den Empfehlungen berücksichtigt.

Dies verdeutlicht, dass eine individuelle Betrachtung der Nährstoffzufuhr und Versorgungslage erforderlich ist, auch wenn eine energieumsatzgerechte und ausgewogene Ernährung das Erreichen der Zufuhrempfehlungen grundsätzlich ermöglicht [4, 17]. Zudem lassen Ernährungsprotokolle, bei denen der Verzehr nur 3–7 Tage protokolliert wird, keine valide und reliable Erfassung der individuellen, habituellen Nährstoffzufuhr zu. Es können lediglich Verzehrgewohnheiten und Lebensmittelpräferenzen auf individueller Ebene sowie eine Schätzung der mittleren Nährstoffzufuhr innerhalb der untersuchten Kohorte vorgenommen werden. Aufgrund individuell unterschiedlicher Resorptionsraten (z. B. auch durch Veränderungen der Bioverfügbarkeiten durch unterschiedliche Lebensmittelzusammensetzung oder Zubereitung; [19]) kann zudem trotz vergleichbarer Mikronährstoffzufuhr eine unterschiedliche Versorgungslage vorliegen.

Zusammenfassend gelten als im Versorgungszustand potenziell kritische Nährstoffe bei Sporttreibenden Eisen (v. a. bei Athletinnen und in Ausdauersportarten), Calcium (v. a. bei Nachwuchsathlet*innen und sich vegan ernährenden Sporttreibenden), Zink (v. a. bei sich vegetarisch ernährenden Sporttreibenden und Heavy-Sweatern^1^), Natrium (v. a. „heavy salty sweater“^2^,^3^) sowie Vitamin D (da die nutritive Zufuhr nur eine untergeordnete Rolle spielt: v. a. bei Sporttreibenden aus Indoorsportarten; [4, 8]).

## Risikofaktoren für Mikronährstoffdefizite im Sport

Sporttreibende haben demnach nicht *per se* einen erhöhten Bedarf an Mikronährstoffen, der nicht über die Ernährung zu decken wäre. Bei ausgewogener Ernährung und nährstoffschonender Zubereitung führt die in der Regel höhere Lebensmittelaufnahme zur Deckung des erhöhten Energiebedarfs auch zu einer höheren Mikronährstoffaufnahme, sodass potenzielle Mehrbedarfe auch mit natürlichen Lebensmitteln (Food-First-Strategie) gut abgedeckt werden können. Dagegen ist insbesondere dann eine kritisch-niedrige Mikronährstoffzufuhr zu beobachten, wenn Sportler*innen z. B. aufgrund von Gewichtsreduktionsambitionen eine unzureichende Energie- und damit Lebensmittelzufuhr aufweisen.

Als Risikogruppe für die Entwicklung eines Eisenmangels sind insbesondere weibliche Ausdauersportlerinnen zu betrachten. Zum Eisenmangel im Ausdauersport können u. a. die sogenannte Marschhämolyse, gastrointestinale Mikroblutungen, belastungsinduzierte Anstiege des Hepcidinspiegels und bei Athletinnen menstruationsbedingte Eisenverluste beitragen [20].

Auch eine suboptimale oder defizitäre Vitamin-D-Versorgung ist bei Athlet*innen mit nennenswerter Prävalenz zu beobachten [21]. Sportler*innen aus Hallensportarten wie Schwimmen oder Turnen sowie Athlet*innen mit dunkler Hautfarbe, hohem Körperfettanteil oder verstärkten UV-Schutzmaßnahmen weisen ein erhöhtes Risiko für eine suboptimale Vitamin-D-Versorgung (30–< 50 nmol/L 25-Hydroxyvitamin D im Serum) oder einen Vitamin-D-Mangel (< 30 nmol/l) auf [8]. Ein suboptimaler Vitamin-D-Status ist aufgrund der überwiegend endogenen Synthese des Vitamins nur marginal ernährungsbedingt und nicht sportspezifisch.

Inadäquate Zufuhrmengen an Calcium werden insbesondere bei weiblichen (Nachwuchs‑)Athletinnen sowie Sporttreibenden mit restriktiver Energiezufuhr oder niedriger Energieverfügbarkeit berichtet.

Magnesium- und Zinkdefizite können durch eine Kombination von unzureichender Zufuhr und sportassoziiert erhöhten Verlusten über Schweiß oder Urin entstehen [22].

Zusammenfassend sind eine restriktive Ernährungsweise mit eingeschränkter Lebensmittelauswahl sowie hohe Belastungsumfänge mit erhöhten Schweißverlusten die wesentlichen Risikofaktoren für eine inadäquate Mikronährstoffversorgung von Sportler*innen.

## Mikronährstoffdefizite und deren Effekte auf Gesundheit und Leistungsfähigkeit

### Natrium

Eine besondere Form eines vorübergehenden Mikronährstoffmangels stellt die sogenannte belastungsinduzierte Hyponatriämie dar. In Deutschland liegt die durchschnittliche Natriumzufuhr weit über den Referenzwerten [23]. Deshalb könnte der natriumbedingte Verlust bei sportlicher Betätigung eine potenziell gesundheitsförderliche Möglichkeit der Ausscheidung überschüssigen Natriums darstellen. In der Regel ist daher der mit dem Schwitzen einhergehende Natriumverlust als unproblematisch einzustufen.

Bei sogenannten Heavy-Salty-Sweatern – erkennbar etwa an deutlich sichtbaren Salzrändern auf der Kleidung und einer hohen Schweißrate – kann es jedoch im Rahmen lang andauernder körperlicher Belastung zu erheblichen Natriumverlusten (Tab. 1) kommen, die eine sportinduzierte Hyponatriämie hervorrufen können [24].

Ein Abfall der Natriumkonzentration im Blut unter 135 mmol/l (Hyponatriämie) wird bei 3–22 % der Teilnehmer*innen von Ausdauersportveranstaltungen festgestellt [25]. Ursächlich für das Auftreten einer sportinduzierten Hyponatriämie ist neben individuellen Risikofaktoren vor allem die Kombination aus gesteigertem Natriumverlust über den Schweiß und einer übermäßigen Aufnahme natriumarmer Flüssigkeiten. Besonders gefährdet sind unerfahrene Sportler*innen, Personen mit einer Belastungsdauer von über 4 h (z. B. bei Marathon‑, Triathlon- oder Ultraausdauerwettkämpfen) sowie Athlet*innen mit einem niedrigen Body-Mass-Index (BMI) und gleichzeitig hoher Flüssigkeitszufuhr [24].

Unspezifische Anfangssymptome wie Übelkeit, Kopfschmerzen oder verminderte Leistungsfähigkeit können von weniger erfahrenen Sportler*innen irrtümlich als Anzeichen einer Dehydratation fehlgedeutet werden. Eine anschließende Aufnahme weiterer natriumarmer Flüssigkeit kann zu einem weiteren Absinken der Natriumkonzentration führen. In der Literatur sind zahlreiche Fälle schwerer Hyponatriämien bei (Massen‑)Sportereignissen dokumentiert, vereinzelt mit tödlichem Ausgang [25]. Trotz der Bedeutung einer angemessenen Natriumzufuhr bei längerer Ausdauerbelastung ist rund 2 Dritteln der Marathonläufer*innen das Risiko einer sportinduzierten Hyponatriämie nicht bekannt [12]. Veraltete Trinkempfehlungen wie „as much as tolerable“ sind nach wie vor weitverbreitet [24].

### Antioxidativ wirksame Mikronährstoffe

Körperliche Aktivität führt über verschiedene Mechanismen zu einer vermehrten Bildung reaktiver Sauerstoff- und Stickstoffspezies („reactive oxygen and nitrogen species“, RONS), auch bekannt als „freie Radikale“ [26]. Für sportlich aktive Personen ist daher eine bedarfsgerechte Zufuhr antioxidativ wirksamer Nährstoffe (z. B. Vitamin C, Vitamin E, Beta-Carotin) essenziell.

Studien zeigen jedoch, dass eine Supplementierung mit Vitamin C und Vitamin E über die Zufuhrempfehlungen hinaus potenziell negative Effekte bei Freizeitsportler*innen haben kann, zum Beispiel auf die Trainingsanpassung und Leistungsentwicklung oder auf Surrogatparameter (Messgrößen, von denen indirekt auf die Zielparameter geschlossen wird) des gesundheitlichen Nutzens [27, 28].

Ursächlich hierfür scheint die gesicherte Rolle reaktiver Spezies (RONS) für die muskuläre Trainingsanpassung und die mitochondriale Biogenese zu sein [29]. Infolge körperlicher Aktivität werden zudem eine gesteigerte endogene antioxidative Kapazität [30] sowie die Zunahme antioxidativer Enzymaktivitäten (z. B. Glutathionperoxidasen, Superoxiddismutasen, Catalasen) beobachtet. Bereits nach wenigen Trainingseinheiten sowie bei jugendlichen Sportler*innen lassen sich verstärkte endogene antioxidative Schutzmechanismen nachweisen [31].

Aufgrund dieser sportassoziiert hochregulierten körpereigenen antioxidativen Schutzmechanismen könnten die Zufuhrempfehlungen für die Allgemeinbevölkerung auch für Sportler*innen trotz erhöhten Bedarfs ausreichend sein. Um das Erreichen der Referenzwerte für Vitamin C, Vitamin E und Beta-Carotin sicherzustellen, wird sportlich aktiven Personen aus heutiger wissenschaftlicher Sicht eine ausgewogene Ernährung mit reichlich antioxidativen Lebensmitteln empfohlen.

Athlet*innen, die sich aus individuellen Gründen für eine Supplementierung entscheiden, sollten dabei die täglich empfohlenen Höchstmengen von 30 mg Vitamin E und 250 mg Vitamin C in Nahrungsergänzungsmitteln nicht überschreiten [32].

### Vitamin D

Sowohl in der Allgemeinbevölkerung als auch bei Sportler*innen ist ein unzureichende Vitamin-D-Versorgung jahreszeitenabhängig zu beobachten [21]. Eine optimale Vitamin-D-Versorgung ist für Athlet*innen von besonderer Bedeutung, da Vitamin D an zahlreichen essenziellen Prozessen beteiligt ist, wie dem Knochenstoffwechsel sowie der Funktion der Skelettmuskulatur [21]. Es ist noch nicht klar, ob eine Vitamin-D-Supplementierung die sportliche Leistungsfähigkeit verbessert, jedoch scheinen Sportler*innen mit einem Vitamin-D-Mangel davon zu profitieren [33]. Für die Allgemeinbevölkerung ebenso wie für Athlet*innen gelten Hydroxyvitamin-D-Serumwerte von ≥ 50 nmol/l als wünschenswert [21].

### Eisen und Eisenmangelanämie

Durch die gesteigerte Vaskularisierung (Gefäßeinsprossung) sowie den Anstieg des Hämatokrits und der Hämoglobinkonzentration bei sportlicher Aktivität kann sich der Eisenbedarf erhöhen [20]. Blutungen, gastrointestinale Blutverluste sowie Blutverluste des Harntrakts können aufgrund hochintensiver Belastung oder des (regelmäßigen) Gebrauchs nichtsteroidaler antiinflammatorischer Medikamente (NSAID) auftreten und zu einem Eisenverlust beitragen. Eine Eisenmangelanämie wirkt sich durch Reduktion des Sauerstofftransports leistungsmindernd aus.

Möglicherweise ist auch ein nichtanämischer Eisenmangel nachteilig mit der sportlichen Leistungsfähigkeit assoziiert, z. B. aufgrund verringerter oxidativer Kapazität, Beeinträchtigung der mitochondrialen Respiration oder verringerter Trainingsadaptation [20]. Sportler*innen mit nichtanämischem Eisenmangel wird vorerst eine Ernährungstherapie bzw. zu einer eisenreichen Ernährung geraten. Entsprechende Empfehlungen zur eisenreichen Ernährung im Sport wurden bereits erwähnt [20].

Eine nicht ärztlich begleitete Eisensupplementation sollte aufgrund gesundheitlicher Risiken einer Eisenüberversorgung, gastrointestinaler Beschwerden, prooxidativer Effekte sowie kontrovers diskutierter Risiken für kardiovaskuläre Erkrankungen und Krebs nicht empfohlen werden [20].

### Mikronährstoffe zur Prävention von Infektionen bei Sportler*innen

Für Erkrankungen der oberen Atemwege, wie beispielsweise viralen Infektionen, konnte wiederholt ein j‑förmiger Dosis-Wirkungs-Zusammenhang zwischen dem Ausmaß körperlicher Aktivität und dem Erkrankungsrisiko festgestellt werden [34]. Sowohl ein niedriges Aktivitätsniveau als auch eine sehr hohe Belastungsintensität und -dauer gehen demnach mit einem erhöhten Infektionsrisiko einher. Möglicherweise sind hierbei insbesondere weniger gut trainierte Personen betroffen.

Vitamin C und Zink werden häufig zur Prävention und Therapie von Erkältungskrankheiten vermarktet. Eine Cochrane-Analyse konnte jedoch bei täglicher Supplementation von 200 mg Vitamin C keinen signifikanten Einfluss auf die Inzidenz, den Schweregrad oder die Dauer der Erkrankung feststellen (relatives Risiko (RR) 0,97; [35]). Unter extremen Bedingungen (z. B. bei Marathonläufen) ließ sich durch eine entsprechende Zufuhr allerdings eine Verringerung der selbstberichteten infekttypischen Symptome beobachten (RR 0,48; [35]). Ob Vitamin C in diesen Fällen tatsächlich eine günstige Wirkung auf den Verlauf viraler Infekte ausübt, bleibt jedoch unklar. Dennoch deckt sich das Ziel einer bedarfsgerechten Versorgung mit Vitamin C mit den Schlussfolgerungen des Panels der Europäischen Behörde für Lebensmittelsicherheit (EFSA). Demzufolge kann Vitamin C zur Aufrechterhaltung einer normalen Funktion des Immunsystems beitragen [36].

Die Einnahme von Zink in pharmakologischen Dosierungen (≥ 75 mg/Tag) scheint in der Allgemeinbevölkerung sowohl die Häufigkeit als auch die Dauer von Erkältungserkrankungen zu senken – nicht jedoch deren Schweregrad [37].

Allerdings traten bei diesen Dosierungen, die deutlich über der tolerierbaren Obergrenze (sog. Tolerable Upper Intake Level) liegen, in den Interventionsgruppen wesentlich häufiger unerwünschte Nebenwirkungen auf als in den Placebogruppen. Im Falle einer Supplementation sollten daher sowohl potenzielle Nebenwirkungen als auch die empfohlenen Tageshöchstmengen für Nahrungsergänzungsmittel berücksichtigt werden (Vitamin C: 250 mg/Tag; Zink: 6,5 mg/Tag; [32]).

## Empfehlungen für die Beurteilung des Versorgungstatus von Athlet*innen mit Mikronährstoffen

Im Verdachtsfall einer defizitären Versorgung mit Mikronährstoffen ist eine Ernährungsanalyse (z. B. Schätzung der Mikronährstoffzufuhr anhand von Verzehrprotokollen) unzureichend zur Beurteilung des Versorgungszustands. Hierfür sind in der Regel Blut- oder Urinanalysen erforderlich [38]. Zu betonen ist jedoch, dass diese klinischen Erhebungen nicht im Regelfall der Ernährungsbetreuung von Sporttreibenden erforderlich sind und zudem Limitationen in der Spezifität und Sensitivität aufweisen können.

Belastungsinduzierte Verfälschungen von Surrogatparametern sind ebenfalls zu berücksichtigen, beispielsweise falsch-hohe Ferritinwerte nach erschöpfenden Belastungen, da Ferritin als Akutphaseprotein reagiert und einen Eisenmangel maskieren kann.

Vom Angebot individualisierter Mikronährstoffmischungen auf Grundlage wissenschaftlich zweifelhafter Methoden (z. B. Haar‑, Speichel- oder Fragebogenanalyse oder Anamnesegespräch) ist Sporttreibenden dringend abzuraten. Der individuelle Versorgungszustand lässt sich mit derartigen Methoden nicht beurteilen. Fundierte Methoden zur Beurteilung des Ernährungs- und Versorgungszustands sind Tab. 3 zu entnehmen.

**Tab. 3:** Empfohlene Methoden und deren Limitationen zur Erfassung des Versorgungszustandes von Athlet*innen mit Mineralstoffen nach [38]

	Biochemische Marker des Status	Limitationen und Vorsichtsmaßnahmen
*Vitamine*		
Vitamin A	Plasma-Retinol	Beeinflusst durch Leberspeicher; bei Surrogat RBP durch Entzündungen und Mangelernährung
Vitamin D	Serum 25(OH)D	Kontroverse Grenzwerte, mehr Forschung benötigt
Vitamin E	Serum Alpha-Tocopherol	–
Vitamin K	Plasmathrombinzeit	–
Vitamin B1 (Thiamin)	ETK-AC, Urin-Thiamin	ETK-AC und Urin-Thiamin reflektieren verschiedene Aspekte des Status
Vitamin B2 (Riboflavin)	EGR-AC, Urin-Riboflavin	Urin-Riboflavin empfindlicher als Serum‑/Plasma-Riboflavin
Vitamin B3 (Niacin)	Urinäre Metaboliten, NMN, 2PY	Kein funktioneller Körperspeichertest verfügbar, NMN und 2PY sinken vor Symptomen
Vitamin B6	Plasma-PLP, weitere: 4‑PA im Urin, EAST-AC und EALT-AC	Mehrere Marker für beste Bewertung erforderlich
Vitamin B12 (Cobalamin)	Serum/Plasma-Cobalamin, Holo-TC II, Serum/Plasma-MMA, Plasmahomocystein	Kein Goldstandard, mehrere Tests für genaue Bewertung nötig
Vitamin C	Nüchtern-Plasma/Serum-Ascorbinsäure, Leukozyten-Ascorbinsäure	Ungenaue Grenzwerte für Mangel, kein Mehrbedarf bei Athlet*innen, Geschlechtsunterschiede
Biotin	Biotin im Urin, Biotin-Metaboliten	Limitierte Informationen bei Athlet*innen
Cholin	Plasma-Cholin, Phosphatidylcholin	Plasma-Cholin nicht sensitiv
Folat	Serum/Plasma-Folat	–
Pantothensäure	Pantothensäure im Urin	–
*Mineralstoffe*		
Calcium	24-Stunden-Urin, erster Morgenurin (Nachturin)	Beeinflusst durch Serumcalcium, Nahrungsmittel, Vitamin-D-Mangel
Chrom	Urin-Chrom, Plasma-Chrom	Analytische Probleme bei Plasma-Chrom
Eisen	Serumferritin (↓ Stadium I), Transferrinsättigung, Serum-Eisen-Transferrin-Rezeptor, Zink-Protoporphyrin, Hämoglobin, Hämatokrit, mittleres korpuskuläres Volumen	Hohe biologische/diurnale Variation, analytische Fehler, Abweichungen in Höhenlagen
Jod	24-Stunden-Urin, TSH, T3, T4, Schilddrüsen-Antikörper	Logistische Schwierigkeiten bei Athlet*innen, Spoturin nur in Populationen aussagekräftig
Kalium	24-Stunden-Urin, zweiter Morgenurin	–
Magnesium	Serum-Magnesium, RBC-Magnesium, ionisiertes Magnesium in Serum und RBC, Magnesium im Urin	Tests ungenau, z. T. geringe Sensitivität und Spezifität, umständlich, kein routinemäßiger Einsatz
Natrium	24-Stunden-Urin, zweiter Morgenurin	–
Phosphor	Serum-Phosphor	Niedrige Sensitivität und Spezifität
Selen	Serum-Selen	Geringe Sensitivität, unsichere Referenzbereiche, abhängig vom Selengehalt der Böden
Zink	Serum-Zink, Urin- und Haarkonzentrationen	Nicht zuverlässig, Serum-Zink später Indikator, durch verschiedene Faktoren beeinflusst

*2PY* N-Methyl-2-pyridon-5-carboxamid, *4‑PA* 4-Pyridoxinsäure, *EALT-AC* Erythrozyten-Alanin-Transaminase-Aktivitätskoeffizient, *EAST-AC* Erythrozyten-Aspartat-Transaminase-Aktivitätskoeffizient, *EGR-AC* Erythrozyten-Glutathion-Reduktase-Aktivitätskoeffizient, *ETK-AC* Erythrozyten-Transketolase-Aktivitätskoeffizient, *Holo-TC II* Holotranscobalamin II, *MMA* Methylmalonsäure, *NMN* N′-Methylnicotinamid, *PLP* Plasma-Pyridoxal-5′-phosphat, *RBC* rote Blutkörperchen (Red Blood Cells), *RBP* Retinol-bindende Proteine, *T3* Triiodthyronin, *T4* Thyroxin, *TSH* Thyreoidea-stimulierendes Hormon

## Fazit

Bei energiebedarfsdeckender Ernährung erreichen Sporttreibende meist eine ausreichende Mikronährstoffzufuhr. Dennoch können sportartspezifische Faktoren – wie hohe Schweißverluste, restriktive Ernährungsformen oder eingeschränkte Lebensmittelauswahl – zu kritischen Versorgungslagen führen.

Besonders relevant sind potenzielle Defizite bei Eisen, Calcium, Zink, Natrium und Vitamin D. Therapeutische Mikronährstoffgaben sind nur bei nachgewiesenem Mangel und Supplemente nur bei unzureichender Zufuhr und erhöhtem Risiko für einen inadäquaten Versorgungszustand sinnvoll. Entscheidend für die ausreichende Mikronährstoffversorgung sind eine bedarfsgerechte Lebensmittelauswahl, nährstoffschonende Zubereitung und gegebenenfalls individuelle Ernährungsberatung mit entsprechenden diätetischen Maßnahmen.

Mikronährstoffdefizite bei Sportler*innen resultieren häufig nicht aus sportartspezifischen Bedarfssteigerungen, sondern eher aus restriktiver Ernährung, inadäquater Energiezufuhr und erhöhten Verlusten (z. B. über Schweiß oder Blutungen). Besonders gefährdet sind Ausdauersportlerinnen (z. B. für Eisenmangel), Sporttreibende aus Indoorsportarten (Vitamin D), Athlet*innen mit restriktiver Diät (Calcium, Magnesium, Zink) sowie Heavy Salty Sweater (Hyponatriämie).

Eine pauschale Supplementierung ist weder grundsätzlich notwendig noch risikofrei. Eine übermäßige Supplementierung – etwa mit den Vitaminen C und E – kann zudem Trainingsadaptationen und somit die Leistungsentwicklung beeinträchtigen.

Die Diagnostik von Mikronährstoffdefiziten bei Sportler*innen erfordert valide Verfahren wie Laboranalysen von Blut- oder Urinproben. Eine ausgewogene Ernährung gemäß Food-First-Prinzip sowie gezielte Diagnostik bei Risikogruppen bilden die Basis für eine adäquate Mikronährstoffversorgung im Sport.

## Footnotes

1 Heavy-Sweater: Sporttreibende mit überdurchschnittlich hoher Schweißrate, wobei die Schweißrate während des Sports intraindividuell stark variiert.

2 Salty Sweater: Sporttreibende mit einem überdurchschnittlich hohen Natriumverlust über den Schweiß, i. d. R. über 1500 mg/Liter. Salty Sweater lassen sich an ausgeprägten Salzrändern an der Sportbekleidung und auf der Haut identifizieren.

3 Heavy Salty Sweater: Sportreibende mit einer überdurchschnittlich hohen Schweißrate und gleichzeitig überdurchschnittlich hoher Natriumkonzentration im Schweiß.
